# Heroes in motion – a six-year quality report and patient evaluation of a real-world exercise therapy program in pediatric oncology

**DOI:** 10.3389/fped.2026.1819559

**Published:** 2026-06-09

**Authors:** Carolin Ohnmacht, Alina Kunder, Sina Benz, Tobias Feuchtinger, Alexander Puzik

**Affiliations:** 1 Department of Pediatric Hematology and Oncology and Stem Cell Transplantation, Children’s Hospital, Medical Center - University of Freiburg, Faculty of Medicine, University of Freiburg, Freiburg, Germany; 2 Department of Sport and Sport Science, University of Freiburg, Freiburg, Germany

**Keywords:** exercise therapy, family-centered, patient satisfaction, pediatric oncology, program implementation, supportive care

## Abstract

**Introduction:**

Rising survival rates in pediatric oncology underscore the importance of interventions that support short- and long-term bio-psycho-social health. Exercise therapy is now recognized as a key component of tertiary prevention and provides survival benefits. In 2020, the “Heroes in Motion” (HIM) program was established at the Department of Pediatric Hematology and Oncology in Freiburg to provide exercise interventions for patients and their families. This real-world service-based evaluation summarizes six years of program implementation, monitoring, and quality improvement, including patient- and parent-reported satisfaction data.

**Methods:**

This multi-method evaluation integrates (a) quantitative data on exercise therapy details combined with structured program documentation and continuous process monitoring (2020–2025) and (b) findings from two satisfaction surveys conducted in 2020 and 2025.

**Results:**

A total of 457 patients (age range: 1–24 years) participated in the HIM program over six years. During the evaluation period, 3.860 inpatient and 813 outpatient sessions were completed. Participation covered diverse diagnoses, with increasing family involvement following the introduction of the family-centered outpatient exercise program in 2023. Satisfaction remained consistently high, with 74% (*n* = 14) in 2020 and 81% (*n* = 13) in 2025 reporting being very satisfied. Participants highlighted individualized care, motivation, and perceived bio-psycho-social benefits. Across all age groups, demand for more frequent sessions and improved exercise facilities (e.g., an exercise room) was consistently expressed.

**Discussion:**

The HIM program proved feasible, safe, and well accepted in a real-world pediatric oncology setting. Ongoing structural development and increasing family involvement emphasize the relevance of exercise therapy within supportive care. Future efforts will focus on establishing a multimodal, family-centered program as part of routine clinical practice.

## 1 Introduction

Regular physical activity is a key determinant of bio-psycho-social health and is defined as any bodily movement produced by skeletal muscles that increases energy expenditure above basal metabolic rate (1). It supports physical fitness, stress regulation and mental health by improving mood, sleep quality, and self-perception, and by reducing symptoms of anxiety and depression (2–6). In childhood cancer, disease- and treatment-related restrictions often lead to a marked decline in physical activity among patients and their families (7–9). During inpatient stays, activity levels of young patients (mean age 12.2 ± 4.7 years, *n* = 125) may decline by up to 91%, with nearly half spending less than one hour per day out of bed (10). Such inactivity increases the risk of long-term adverse outcomes, including cardiovascular disease, chronic pain and cancer-related fatigue (11–14).

As survival rates for childhood cancer now exceed over 80% (15), promoting an active lifestyle has become an essential component of tertiary prevention. Evidence from adult oncology shows that regular physical activity combined with healthy nutrition can reduce cardiometabolic risk and improve overall quality of life in survivors (16–18). The German AWMF-S2k guideline (19) provides evidence- and consensus-based recommendations for integrating physical activity during and after cancer treatment, and the UN Convention on the Rights of the Child and SIOPe emphasize every child's right to movement (20–22). Systematic reviews demonstrate that targeted exercise programs can reduce fatigue and enhance health-related quality of life in children with cancer (23–25). Exercise is defined as planned, structured, and repetitive physical activity aimed at improving physical fitness (26). Individually supervised interventions appear particularly effective and promote physiological and psychological benefits, including improved treatment tolerance (27, 28). Recent evidence suggests that targeted sensorimotor training may help reduce or prevent peripheral neuropathy in children and adolescents undergoing neurotoxic chemotherapy (29).

Reduced physical activity also affects parents of children with cancer, who report increased sedentary behavior, new physical complaints, and a desire for structured opportunities to remain active (9). Parents play a central role in shaping their children's physical activity behavior, for example by acting as role models, being active themselves or together with their children (30, 31).

Despite growing evidence, sustainable implementation of exercise therapy in routine pediatric oncology remains challenging, and long-term real-world data on feasibility, acceptance, and program quality, including safety, are limited. The "Heroes in Motion" (HIM) program, a structured exercise therapy initiative, has been implemented and continuously developed since March 2020 at the Department of Pediatric Hematology and Oncology in Freiburg, Germany. At that time, 13 of 60 pediatric oncology centers nationwide offered exercise therapy. Today, more than 40 centers have implemented similar programs and are part of the Network Active Onco Kids (NAOK), a nationwide initiative funded by the German Cancer Aid that supports children and adolescents with cancer in accessing individualized physical activity opportunities and assists pediatric oncology centers in developing and expanding structured exercise programs(32).

This work presents a six-year service-based evaluation of the HIM program, including its implementation and ongoing quality improvement measures. Feasibility, acceptance, and safety were assessed, and key enablers, prerequisites, and barriers to implementation were identified.

## 2 Methods

### 2.1 Design and setting

This quality report presents a six-year multi-methods evaluation of the HIM program and is designed as a real-world, service-based evaluation using routinely collected data. Consistent with previous real-world program evaluations in pediatric oncology, data were derived from multiple sources, including routine therapy-related documentation, structured program records, and two satisfaction surveys. The evaluation integrates (a) quantitative data fromroutine therapy-related documentation, structured program documentation, and continuous process monitoring collected between March 2020 and December 2025, and (b) qualitative and quantitative data from two voluntary satisfaction surveys conducted in 2020 (July 13–30) and 2025 (January 22–March 7).

The report follows a descriptive, retrospective observational design based on routinely collected clinical data. Analyses were limited to descriptive statistics; no inferential statistical analyses or hypothesis testing were performed.

Contextual factors related to program implementation; specifically prerequisites, enablers, and barriers, were identified through structured program documentation and continuous process monitoring. This data-driven approach aligns with previous real-world evaluations in pediatric exercise oncology, where implementation factors are derived empirically rather than conceptually (33–35).

The HIM program is embedded in routine clinical care at a tertiary care pediatric oncology center. Its implementation is shaped by existing clinical structures, staffing resources, and available facilities. As one of the largest pediatric oncology and stem cell transplantation centers in Germany, the Department of Pediatric Hematology and Oncology in Freiburg treats approximately 80–100 children and adolescents with cancer each year, including 30–40 patients undergoing allogeneic hematopoietic stem cell transplantation (HSCT).

### 2.2 The HIM program

The HIM program provides individualized, and supervised exercise therapy for patients (usually ≥2 years) with hematological and oncological diseases, as well as for their families. Upon request, younger children or patients with non-malignant disorders (e.g., diabetes, gastrointestinal disorders, malformations) were occasionally included (Table 1). The program aims to counteract physical inactivity, maintain functional capacity, prevent or reduce treatment-related side effects, and promote psychosocial well-being. Following intensive treatment phases such as hematopoietic stem cell transplantation (HSCT), the program supports a safe and gradual reintegration into physical activity. Family involvement and community-based exercise events in cooperation with regional sports clubs are integral components to promote long-term engagement in physical activity. Teaching activites, networking and scientific projects are conducted in parallel but are not part of this report.

**Table 1:** Participant characteristics (*n* = 457). Results in *n* (%), MD ± SD and range.

Patient characteristics	*n* (%)	MD ± SD	Range
Age (years)		9 ± 5.5	1–24
Sex			
Male	289 (63)		
Female	168 (37)		
Diagnostic entities			
Leukemias	158 (35)		
Solid tumors	112 (24)		
Lymphomas	59 (13)		
CNS tumors	25 (6)		
Hematologic disorders	43 (9)		
Immunologic disorders	36 (8)		
Non-malignant diseases	24 (5)		

This section is reported in accordance with the TIDieR (Template for Intervention Description and Replication) reporting guidelines (36).

Since March 2020, HIM has been implemented as individualized inpatient exercise therapy within the NAOK and was expanded by an outpatient exercise program since 2023. During the evaluation period (2020–2025) HIM was delivered by one to two certified exercise therapists with expertise in pediatric oncology under continuous clinical supervision. Training frequency ranged from three to five sessions per week in the inpatient setting and two sessions per week in the outpatient setting, with total duration depending on the individual clinical course.

In the inpatient setting, exercise therapy was delivered flexibly and condition-dependently rather than through fixed appointments. Sessions were conducted individually or in small groups at the bedside, or on the pediatric oncology ward during weekdays, with optional home-based continuation via individualized exercise plans (Supplementary Material). Exercise therapists therefore performed regular bedside rounds and offered participation based on the child's daily medical condition and well-being. Participation was voluntary and could be declined at any time. Non-participation was most commonly related to medical procedures, reduced general condition, fatigue or sleep, lack of motivation, or absence from the room. This approach reflects real-world clinical practice, where offered contacts do not consistently translate into completed sessions.

Delivery was adapted to staffing resources and clinical workload; weekend sessions were not routinely provided. HIM was individually tailored from diagnosis onward in accordance with German clinical practice guidelines [AWMF S2k “Promotion of Exercise and Exercise Therapy in Pediatric Oncology”; (19)]. Content targeted strength, endurance, coordination, flexibility, and sensorimotor skills, ranging from play-based activities in younger children to structured training in adolescents.

The outpatient program primarily targets post- HSCT patients and their families and was conducted in the exercise room of the parents’ house adjacent to the Children's Hospital Freiburg, operated by the “Förderverein für krebskranke Kinder e.V.”

Sessions followed a standardized structure (warm-up, main exercise phase, cool-down) and included play-oriented and functional exercises focusing on strength, endurance, coordination, and relaxation, informed by established pediatric oncology exercise therapy models (8, 19, 33). Family members were actively involved to promote shared physical activity, enhance enjoyment of movement, and provide relief from the clinical routine. Community-based exercise offerings (“sport action days”) were additionally organized.

Content and intensity were continuously adapted to medical status, treatment phase, developmental level, and physical and psychological resilience. Training was modified or paused in the presence of contraindications such as severe thrombocytopenia or anemia, fever, infections, or acute symptoms (13, 19).

The HIM program underwent continuous refinement throughout the evaluation period, including increased training frequency, introduction of the outpatient component, and expansion of community-based exercise events. A temporary reduction in staffing capacity between October 2024 and September 2025 led to reduced service availability, underscoring the dependence of implementation on staffing resources.

No predefined number of sessions per patient per week was established; actual delivery was determined by clinical feasibility, staffing capacity, and the patient's daily condition. From October 2025 onward, the program resumed full capacity with two exercise therapists providing services five days per week. Participation was voluntary, donation-funded, and embedded within a research network. The exercise therapy unit has been certified by the German Society for Pediatric Oncology and Hematology (GPOH) since 2025.

### 2.3 Data sources and analysis

Data for this evaluation were derived from three main sources: (1) routinely collected therapy-related documentation of the HIM program, (2) structured program documentation and continuous process monitoring, and (3) survey-based assessments of patient and parent satisfaction. These data sources were used to evaluate feasibility, safety, and acceptance, and to identify contextual factors influencing program implementation.

Contextual factors (i.e., prerequisites, enablers, and barriers) were not assessed using a predefined instrument. Instead, they were derived inductively from routine documentation and survey data using a data-driven approach. This methodology aligns with real-world evaluation practices in pediatric exercise oncology, where implementation-relevant factors are typically identified from observational and routine clinical data rather than through standardized tools (33–35).

#### 2.3.1 Routine monitoring of exercise therapy (2020–2025)

Aggregated data were extracted from routine HIM program documentation. After initial patient contact, individuals were added to the pseudonymized documentation list and included in the analysis. The program underwent continuous refinement, accompanied by expanded documentation procedures. Each session conducted between March 2020 and December 2025 was recorded in a database capturing demographics, diagnosis, prior sports experience, date, duration, session content, family involvement, and free-text notes (e.g., reasons for non-participation, interruptions, and contextual factors such as medical limitations, fatigue, or organizational constraints).

No dedicated instrument was used to assess barriers or enablers. Instead, these factors were retrospectively derived from routine documentation by screening structured fields and free-text entries and summarizing recurring themes descriptively. Accordingly, barriers and enablers reflect real-world conditions and documented implementation-related circumstances rather than systematically elicited constructs.

We assessed the frequency and duration of inpatient and outpatient sessions, as well as patient and family participation across predefined time periods. These indicators were used to evaluate program utilization and feasibility. Feasibility was defined as a multidimensional construct encompassing practical implementation (recruitment, participation, and adherence), safety, acceptability of the HIM-program, and contextual feasibility, including integration into clinical workflows and dependence on staffing and organizational resources (37). Descriptive analysis included percentages (%), median (MD) ± standard deviation (SD), and range.

#### 2.3.2 Patient and parent satisfaction surveys (2020 and 2025)

Patient and parent satisfaction with the HIM program was assessed using two anonymous, voluntary, paper-based surveys conducted in German in 2020 and 2025 (Supplementary Material). All eligible patients and caregivers who met the respective inclusion criteria and were present on the pediatric oncology ward during the defined survey periods were invited to participate. Participation was voluntary, and all questionnaires were returned anonymously via a collection box.

##### 2.3.2.1 Satisfaction survey 2020 (July 13–30, 2020)

The initial survey targeted pediatric patients aged ≥3 years who had participated in at least one HIM session and had sufficient German language proficiency. An internally developed questionnaire consisting of eight predominantly quantitative items (rating scales) with open-ended questions was used. No parent survey or age-specific patient versions were included in 2020, as age-differentiated instruments had not yet been developed.

##### 2.3.2.2 Satisfaction survey 2025 (January 22–March 7, 2025)

The revised survey expanded the target population to include both patients (≥2 years) and parents. Eligibility required participation in at least three HIM sessions and sufficient German language proficiency. In contrast to 2020, age-specific patient questionnaires were conducted in 2025 (2–6, 7–11, ≥12 years), alongside a separate standardized parent questionnaire. The instrument combined closed Likert-scale items (1–4) with open-ended questions.

##### 2.3.2.3 Data analysis and comparability

To ensure methodological transparency, qualitative data from open-ended survey responses and routine documentation were analyzed using reflexive thematic analysis as described by Braun et al. (38). This iterative analytic process included familiarization with the data, systematic coding, and subsequent theme development. Qualitative data from both survey time points (2020 and 2025) were analyzed jointly to identify overarching patterns across patient and parent perspectives. Due to methodological differences between the two surveys (absence of age-specific versions in 2020, inclusion of parents only in 2025, and questionnaire refinement), comparisons between 2020 and 2025 were limited to descriptive patient-reported data.

All survey instruments are provided in the Supplementary Material (German Version, translation available upon request).

## 3 Results

### 3.1 Feasibility, sustainability and safety of exercise therapy

#### 3.1.1 Cohort

Between March 2020 and December 2025 (2,217 days), a total of 457 individual pediatric patients participated in inpatient and/or outpatient exercise therapy sessions. Most participants were diagnosed with malignant diseases. Leukemias represented the largest subgroup (*n* = 158, 35%), followed by solid tumors (*n* = 112, 24%) and lymphomas (*n* = 59, 13%). Central nervous system (CNS) tumors accounted for 6% (*n* = 25); benign CNS tumors were not included, as these patients were treated on the neuropediatric ward rather than the oncology ward. Hematologic (*n* = 43, 9%) and immunologic disorders (*n* = 36, 8%) were less frequent (Table 1).

#### 3.1.2 Exercise therapy details and surveillance

##### 3.1.2.1 Inpatient sessions

A total of 6,353 inpatient exercise sessions were offered between 2020 and 2025, of which 3,860 sessions (61%) were completed. The remaining 2,493 sessions were not conducted due to medical procedures, reduced general condition, lack of motivation, sleep, or absence of the child (e.g., being outside the ward). Reasons were documented in clinical notes but were not systematically categorized, precluding a reliable quantification of individual causes. A standardized documentation system has been implemented since 2026 to enable more detailed analyses in future evaluations.

The median duration of completed session was 31 min (range: 5–96 min). Systematic documentation of family involvement began in 2024; since then, parents participated in 643 and siblings in 141 inpatient sessions (Table 2).

**Table 2:** Annualized details on exercise therapy sessions from 2020 to 2025. Results in *n* (%), MD ± SD or range.

Cohort	2020	2021	2022	2023	2024	2025
Number of patients	67	101	95	119	115	162
Age (years)	10 ± 4.8	10 ± 5.1	10 ± 5.4	9 ± 5.8	8 ± 5.6	9 ± 5.8
Male, *n* (%)	30 (45)	57 (56)	56 (59)	79 (66)	80 (70)	97 (45)
Female, *n* (%)	37 (55)	44 (44)	39 (41)	40 (34)	35 (30)	65 (55)
Session details for inpatients						
Offered sessions	412	872	906	1,419	1,548	1,196
Completed sessions	212	482	681	916	917	652
Duration (min)	25 ± 16.9	30 ± 16.0	30 ± 16.1	30 ± 15.1	30 ± 13.3	30 ± 13.6
Duration range (min)	5–75	5–120	5–95	5–120	5–75	5–90
Parent sessions	–	–	–	–	332	311
Sibling sessions	–	–	–	–	83	58
Session details for outpatients						
Number of patients	–	–	–	29	24	27
Male, *n* (%)	–	–	–	16 (55)	18 (75)	16 (59)
Female, *n* (%)	–	–	–	13 (45)	6 (25)	11 (41)
Completed sessions	–	–	–	352	247	214
Session duration (min)	–	–	–	30 ± 11.1	30 ± 7.2	45 ± 13.3
Duration range (min)	–	–	–	30–60	30–45	30–90
Parent sessions	–	–	–	58	126	66
Sibling sessions	–	–	–	105	69	19
Availability and feasibility						
Days with exercise therapy/year	73	144	175	213	218	169
Total sessions/day	2.9	3.3	3.9	6.0	5.3	5.1

##### 3.1.2.2 Outpatient sessions and family-centered exercise therapy

Though further study is needed with respect to interpersonal influences and parents' roles specifically, the impact of the family system on physical activity behaviour and intervention uptake is compelling.

Between 2023 and 2025, a total of 813 outpatient exercise therapy sessions (range: 30–65 min) were conducted for HSCT patients and their families in the exercise room of the parents’ house adjacent to the Children's Hospital Freiburg. Sessions were documented per participating child. If multiple children attended, each participation was counted as an individual session. Only completed sessions were systematically recorded. Planned but non-completed sessions (e.g., due to absence) were not consistently documented, and therefore no data on offered sessions or reasons for non-completion are available.

Family involvement increased following the introduction of the structured outpatient exercise therapy program in 2023 (twice weekly). Since then, parents participated in 250 sessions and siblings in 183 (Table 2). A weekly parent-only exercise group was piloted in July 2024 (five sessions, 16 participants) but was discontinued due to staffing limitations.

##### 3.1.2.3 Integration of community-based exercise events (“sport action days”)

Since September 2023, the program has been supplemented by regular community-based exercise events (“sport action days”) for patients and their families. These events were conducted in cooperation with regional partners, including Deutscher Behindertensportverband, Badischer Behindertensportverband, Hobby-Horsing FT Freiburg, Badische Sportjugend, Badischer Sportbund and INITIATIVE e.V. The sport action days provided structured, group-based exercise opportunities in addition to routine inpatient and outpatient sessions and were fully integrated into the HIM program. During the evaluation period, nine sport action days were conducted, with participation from 65 patients, 69 parents, and 42 siblings. Three events were held within the hospital, while six took place in community settings (e.g., the parents’ house adjacent to the Children's Hospital Freiburg). Participation in these events is included in Table 2.

##### 3.1.2.4 Adverse events

Across 992 documented days with exercise therapy (Table 2), no serious adverse events occurred during the entire six-year period of exercise therapy program.

### 3.2 Satisfaction surveys (2020 and 2025)

During the 2020 survey period, 37 patients participated at least once in the HIM program. Of these, 18 patients (49%) were not eligible for the satisfaction survey due to being unreachable (78%, *n* = 14), reduced general condition (11%, *n* = 2), or death (11%, *n* = 2). Nineteen eligible patients or caregivers received the questionnaire, and all returned it (response rate 100%).

In 2025, 46 patients completed at least three HIM sessions during the survey period. Of these, 23 patients and caregivers were successfully contacted and received the age-specific patient questionnaire and the parent questionnaire. Sixteen completed questionnaires were returned (response rate 70%). Non-participation among eligible families was due to absence during the survey period (*n* = 12), language barriers (*n* = 7), or reduced general condition (*n* = 4).

These numbers reflect the eligible patients during the defined survey windows and do not represent the full HIM cohort of 457 patients across six years.

**Figure 1:**
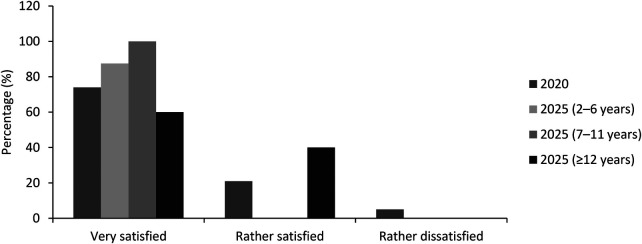
Overall satisfaction with exercise therapy (2020, *n* = 19 and 2025, *n* = 16).

Across both survey years, satisfaction with exercise therapy was consistently high. In 2020, 95% of respondents reported being satisfied, based on 74% (*n* = 14) who were “very satisfied” and 21% (*n* = 4) who were “rather satisfied”. Only one participant reported being “rather dissatisfied” due to insufficient session frequency. In 2025, 81% (*n* = 13) of respondents were “very satisfied” and 13% (*n* = 2) were “rather satisfied” with the HIM program (Figure 1). Both surveys indicated high satisfaction with the structure, content, and quality of the program. In 2025, all respondents (100%, *n* = 16) reported a high degree of perceived safety while participating in HIM. Safety was not assessed in 2020.

#### 3.2.1 Patient and parent perspectives (qualitative findings)

Qualitative data from open-ended survey responses and routine documentation provided additional insight into patient and parent experiences with the HIM program. Across both survey time points, three main thematic clusters emerged: (1) perceived benefits of the program, (2) desire for increased access, and (3) contextual facilitators and barriers to participation.

##### 3.2.1.1 Perceived benefits

Across both surveys, patients and parents consistently described the program as enjoyable, motivating, and beneficial for both physical and emotional well-being. In 2020, patients emphasized activating and mood-enhancing effects, reflected in representative statements such as “*you could always laugh”* and “*it helps to activate circulation and mobilize the body”.* In 2025, parents highlighted both psychosocial and functional benefits. One parent reported: “*Sports therapy is a great success for him, success in ‘this is something I can still do!’ For me, it is like ‘balm for the soul’ to see my son having fun”.* Others described functional improvements: “*Sports therapy helped our child much more than physiotherapy to regain mobility”.* Overall satisfaction was frequently linked to individualized care, therapist competence, and visible positive effects on the child's well-being.

##### 3.2.1.2 Desire for increased access

A recurring theme across both survey years was the wish for more frequent and extended access to exercise therapy. In 2020, patients expressed a desire for daily sessions (e.g., “*It would be nice to be able to participate every day”*). Similarly, in 2025, parents emphasized the need for program expansion, stating that it “*urgently needs to be expanded and supported”,* and expressed a preference for more frequent and longer sessions, including daily provision.

##### 3.2.1.3 Contextual enablers and barriers

Structural and environmental factors were identified as both facilitators and barriers to participation. In 2020, patients reported limited space and the absence of a dedicated therapy room as barriers: “*a special room with more space and freedom of movement would be desirable*”. Parents also noted that sessions conducted in shared or transitional spaces hindered participation.

In 2025, parents highlighted individualized care and therapist-related factors as key facilitators, describing the program as “*always motivating, friendly, and adapted to the child's current condition”* (parents of a 2–6-year-old patient). The perceived value of the program as strongly emphasized, for example: “*Rarely have we seen such a good program that urgently needs to be further expanded and supported! It gives children a piece of quality of life and security back”* (parents of a 2–6-year-old patient, 2025). At the same time, parents reiterated the need for improved infrastructure and expanded resources, including adequate space and increased program capacity.

Overall, qualitative findings complemented the quantitative results by demonstrating high acceptability, perceived physical and psychosocial benefits, and a consistent demand for increased availability and structural optimization of the program.

#### 3.2.2 Facilitators (enablers) and barriers for implementation

During the course of the program, implementation-relevant factors were categorized into four domains: human resources, spatial resources, equipment, funding, and classified as facilitators or barriers.

- Human resources: Stable, adequately trained exercise therapists were a key facilitator for program continuity. In 2025, staffing reductions represented a barrier, leading to a 29% decrease in inpatient sessions (2024: 917 → 2025: 652; Table 2). Inpatient exercise therapy was temporarily reduced from five to four days per week, and additional parent and sibling offerings were paused.
- Spatial resources/facilities: Lack of a dedicated therapy room was a frequently reported barrier. Sessions in corridor wards reduced privacy, motivation, and participation, especially among adolescents. In contrast, an exercise room in the parents’ house adjacent to the Children's Hospital Freiburg provided a facilitating environment for outpatient sessions.
- Equipment: At program initiation, equipment availability was limited and materials had to be brought in by staff. Over time, the establishment of a dedicated storage area and acquisition of sufficient equipment facilitated routine implementation.
- Integration into clinical workflow: Close coordination with medical and psychosocial teams supported the integration of exercise therapy into daily clinical routines and enhanced feasibility.
- Funding: All sessions were funded through donations; only very few centers in Germany can bill health insurance through individual contracts.
- Integration and governance: Interdisciplinary coordination facilitated implementation throughout the program. Since 2025, HIM has been recognized as a quality-assured program by the German Society for Pediatric Oncology and Hematology (GPOH), requiring structured documentation, trained staff, and defined program components. This certification served as an additional facilitator by formalizing program standards and governance structures.

## 4 Discussion

This six-year quality report demonstrates that the HIM program is feasible, safe, and well accepted in a real-world pediatric oncology setting. As a real-world, service-based evaluation, the findings should be interpreted descriptively and within their specific care context. The high number of completed sessions across diagnostic groups and age ranges underscores the applicability of individualized exercise therapy in adequately resourced settings and its sustainability as part of supportive care. These findings align with previous evidence showing that supervised exercise during cancer treatment is both safe and beneficial in clinical trials (23, 24). Similar real-world evaluations of pediatric oncology exercise interventions have reported high feasibility and acceptance under routine care conditions, supporting the transfer of structured exercise programs into clinical practice (28, 39). From a social-ecological perspective (40), these outcomes reflect the interaction between individual engagement and the structural conditions under which the program is delivered.

The continuous expansion of inpatient, outpatient, and community-based events highlights the program's adaptability and relevance within real-world care structures. The increase in session numbers from 2020 to 2024 reflects successful integration into clinical routines and growing acceptance among patients, families, and staff. The decline in 2025 highlights the vulnerability of exercise therapy programs to staffing fluctuations. At the organizational level of the social-ecological model, this finding emphasizes the dependence of program delivery on stable staffing structures, institutional support, and resource allocation (40), underscoring the need for long-term financing and structural anchoring.

Across the entire period, no serious adverse events occurred, and minor events were rare and manageable through session adaptation. This confirms the high safety of supervised exercise during intensive cancer treatment and is supported by the perceived safety ratings in 2025. These findings are consistent with controlled and observational studies demonstrating the safety of exercise interventions in pediatric oncology populations, even during intensive treatment phases (39). The introduction of a national adverse event registry by NAOK will enable more precise monitoring in the future.

Increasing participation of parents and siblings reflects the recognition that childhood cancer affects the entire family system. At the interpersonal level (40), previous research shows that parents often experience reduced physical activity, stress, and physical complaints during their child's treatment (9). At the same time, shared physical activity can support coping and resilience (30). The strong parental endorsement observed in our program underscores the potential of family-centered exercise therapy in real-world pediatric oncology setting.

This is consistent with growing evidence that parental involvement enhances intervention effects (41) and that children are more likely to be physically active when one or both parents are active (42). Recent pediatric exercise oncology studies highlight the benefits of family-inclusive intervention approaches for adherence and psychosocial outcomes (39). Findings by Erkelenz et al. (43) demonstrate that parental physical activity is not necessarily directly associated with children's moderate-to-vigorous physical activity levels, but is strongly linked to increased participation in sports and lower BMI percentiles in children. This suggests that parental facilitation, such as enabling access to structured exercise programs may be more relevant than parental activity behavior itself. Similarly, Wilk et al. (44) highlight that parental support behaviors (e.g., encouragement, transportation, co-participation) and, importantly, the children's perception of this support are key determinants of their physical activity levels, whereas parental physical activity alone showed no significant direct effect. These findings reinforce the notion that the interpersonal environment, particularly perceived support, plays a central mediating role in shaping children's activity behavior. On the contrary, a recent dyadic analysis in children with brain tumors found a strong positive correlation between parent and child physical activity levels, indicating shared behavioral patterns within families (45). However, only the child's own physical activity was directly associated with improved quality of life outcomes. This suggests that while parental behavior may influence children's engagement, its effects on health outcomes are primarily indirect and mediated through the child's own activity.

These findings highlight the central role of family systems in shaping health behaviors and intervention uptake (41), underscoring the importance of integrating structured, family-centered components into exercise therapy programs. Though further study is needed with respect to interpersonal influences and parents' roles specifically, the impact of the family system on physical activity behavior and intervention uptake is compelling. In the context of pediatric oncology, where children face substantial physical and psychosocial barriers to activity, parents may act less as role models and more as enablers, motivators, and facilitators of participation.

Despite high satisfaction, families repeatedly emphasized the need for improved facilities. Conducting sessions in hallways was perceived as a barrier, particularly by adolescents and parents, echoing previous evidence (9, 28). At the environmental and organizational levels of the social ecological model, physical infrastructure emerges as a key determinant of participation, privacy, and motivation in pediatric supportive care (40). The strong desire for more frequent and longer sessions indicates that current resources do not fully meet patient and family needs and highlight the importance of stable staffing and dedicated therapy space. In this context, group-based exercise sessions may represent a potential strategy to increase efficiency and reach within limited staffing resources. Such formats could allow more patients to benefit from the program simultaneously and may enhance peer interaction and motivation. However, their implementation would require adequate spatial capacity, appropriate patient matching in terms of medical condition and treatment phase, and careful consideration of safety and individualization, which may limit their feasibility in the real-world setting.

The differences in survey response rates between 2020 and 2025 further illustrate how methodological and contextual factors shape evaluation outcomes in real-world settings. The higher response rate in 2020 may be partly explained by the novelty of the program and the shorter, less demanding questionnaire, which likely facilitated participation. In contrast, the 2025 survey involved more complex, age-adapted instruments and required a minimum of three completed sessions, increasing respondent burden. Additional structural barriers—such as patient absence during the survey period and language limitations—further reduced participation in 2025. These factors highlight how accessibility, timing, and survey design influence who is reached in routine care evaluations and underscore the need for standardized, low-threshold assessment procedures to ensure representativeness.

Several methodological aspects must be considered when interpreting the satisfaction data. First, the two surveys differed in structure and inclusion criteria. In 2020, participation required only one completed session, whereas in 2025 at least three sessions were required. This may have introduced selection bias. In addition, only a small proportion of eligible participants responded to the surveys across both time periods, which increases the risk of selection bias and limits representativeness. It is possible that primarily highly satisfied families participated, potentially leading to an overestimation of positive evaluations. At the same time, repeated participation enabled respondents to provide more experience-based assessments of the program. Second, the 2025 survey included age-specific questionnaires and a parent questionnaire, while the 2020 survey used a single instrument for all children aged ≥3 years. These limits direct comparability. Third, contextual changes likely influenced satisfaction ratings. Some participants in the 2025 survey may have experienced the program in earlier years, when the session availability was transiently higher. Their desire for expanded offerings may reflect prior exposure to a more extensive program structure. Additionally, patient numbers nearly doubled following the move to a new hospital building, while staffing was temporarily reduced. Fourth, both surveys relied on internally developed, non-validated questionnaires. Although this approach allowed age-appropriate and context-specific assessment, it limits generalizability and comparability with other studies. These limitations are inherent to real-world evaluations relying on routinely collected clinical data and should be considered when interpreting the findings. The low number of respondents, as well as the time-limited data collection periods due to thesis-related constraints, further limit the robustness of the findings. Accordingly, results should be interpreted with caution, as they may not be fully representative of the broader patient population.

Future refinement of the HIM program will focus on strengthening family-centered modules, expanding outpatient and community-based exercise opportunities, and improving spatial infrastructure. In line with a social-ecological framework, these refinements target multiple levels, including individual engagement, family involvement, and organizational capacity (40). With stable staffing and structural support, HIM is well positioned to evolve into a sustainable, multimodal exercise therapy program embedded in routine pediatric oncology care. As such, this program contributes to the growing body of real-world evidence supporting the implementation of exercise interventions in pediatric oncology.

## 5 Conclusion

The HIM program has proven feasible, safe, and highly valued in a real-world pediatric oncology setting. Its family-centered orientation, individualized approach, and integration into clinical routines represent major strengths. However, sustainable implementation requires stable staffing, adequate facilities, and secure financing. Consistently positive feedback from patients and families underscores the therapeutic and psychosocial relevance of individualized, family-centered exercise therapy during cancer treatment. The increasing participation of parents and siblings further highlights the program's potential to strengthen family resilience and support coping during intensive treatment phases. While HIM may serve as a successful real-world example of supportive therapies in pediatric oncology, there is a major need to create further evidence for the efficacy of such therapies and make multimodal exercise therapy structures available to all young cancer patients. Future refinement should therefore focus on expanding outpatient and community-based components, improving spatial infrastructure, and embedding standardized quality criteria to ensure long-term sustainability and equitable access across care settings.

## Data Availability

The raw data supporting the conclusions of this article will be made available by the authors, without undue reservation.
