# Supplementary material for: Heroes in motion – a six-year quality report and patient evaluation of a real-world exercise therapy program in pediatric oncology
Source: Front Pediatr. 2026 Jun 9;14:1819559. doi: 10.3389/fped.2026.1819559 (PMC13286956; doi:10.3389/fped.2026.1819559)
Supplement: Supplementary file 1 [file Datasheet1.pdf]

frontiers

Supplementary Material

1. Satisfaction survey – 2020

HELDEN IN BEWEGUNG

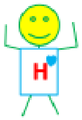

FRAGEBOGEN

Evaluation der Zufriedenheit mit der sporttherapeutischen Versorgung auf der Station v. Pfaundler

HELDEN IN BEWEGUNG

Liebe/r ________________,

Vielen Dank, dass Du Dir für diesen Fragebogen Zeit nimmst!

Seit März 2020 findet an der Klinik für Pädiatrische Hämatologie und Onkologie am Zentrum für Kinder- und Jugendmedizin in Freiburg eine stationäre Sport- und Bewegungstherapie, ergänzend zu bestehenden Angeboten, statt. Wir freuen uns sehr, dass Du am sporttherapeutischen Bewegungsangebot teilgenommen hast! Die folgenden Fragen sind wichtig für uns, um herauszufinden, wie zufrieden Du mit dem Sportangebot während Deines stationären Aufenthaltes gewesen bist. Die Beantwortung der Fragen ist freiwillig. Das Ausfüllen dauert maximal 10 Minuten.

Hinweise zum Ausfüllen des Fragebogens:

- Solltest Du noch zu klein zum Ausfüllen des Fragebogens sein, dürfen Dir natürlich Deine Eltern dabei helfen.
- Wir sind an Deiner persönlichen Meinung in Bezug auf Deine Zufriedenheit interessiert, deshalb gibt es keine „richtigen“ oder „falschen“ Antworten.
- Falls eine Frage weniger auf Dich zutrifft oder es Dir schwerfällt, Dich für eine Antwort zu entscheiden, kreuze bitte die Antwort an, die spontan am ehesten auf Dich zutrifft.
- Bitte markiere die für Dich zutreffenden Kästchen mit einem Kreuz: ☒
- Stellen, an denen wir Dich bitten, etwas aufzuschreiben, sind durch einen Kasten: ☐ gekennzeichnet.
- Der Fragebogen wird anonymisiert ausgewertet, Deine Angaben im Fragebogen können also nicht mit Deinem Namen in Verbindung gebracht werden. Die Angaben werden ausschließlich zur Evaluation der Projektzufriedenheit verwendet.

Mit freundlichen Grüßen

Carolin Ohnmacht (B.A. Sportwissenschaftlerin und Sporttherapeutin)

Vorab möchten wir gerne ein paar allgemeine Informationen über Deine Person erfahren.

1. Wie alt bist Du?
Kreuze an!

❑ 3-5 Jahre

❑ 6-10 Jahre

❑ 11-13 Jahre

❑ 14-17 Jahre

❑ 18 Jahre oder älter

2. Welches Geschlecht hast Du?
Kreuze an!

❑ Junge

❑ Mädchen

Deine Meinung ist uns wichtig. Im folgenden Teil möchten wir gerne erfahren, wie zufrieden Du mit dem sporttherapeutischen Angebot während Deines stationären Aufenthaltes im Allgemeinen gewesen bist.

3. Wie zufrieden warst Du mit ...	sehr zufrieden	eher zufrieden	eher unzufrieden	ziemlich unzufrieden
... dem Sportangebot insgesamt?	❑	❑	❑	❑
... dem Ablauf (z.B. Kontaktaufnahme)?	❑	❑	❑	❑
... den Inhalten (z.B. Übungsauswahl)?	❑	❑	❑	❑
... dem Ausmaß (Häufigkeit, Dauer)?	❑	❑	❑	❑
... der Qualität?	❑	❑	❑	❑
❑ Sonstiges:				

4. Kreuze bitte an:	trifft voll zu	trifft zu	trifft nicht zu	trifft überhaupt nicht zu
Hast Du die Art von Bewegung erhalten, die Du wolltest?	❑	❑	❑	❑
Würdest Du einem Freund/einer Freundin das Sportprogramm empfehlen, wenn er/sie in einer ähnlichen Situation wie Du wäre?	❑	❑	❑	❑

HELDEN IN BEWEGUNG

**5. Bitte bewerte die folgenden Aussagen und kreuze an:**	**trifft voll zu**	**trifft zu**	**trifft nicht zu**	**trifft überhaupt nicht zu**
Das Programm war an meinen aktuellen Gesundheitszustand angepasst	❑	❑	❑	❑
Ich hatte das Gefühl, dass mir der Sport während des stationären Aufenthaltes gutgetan hat	❑	❑	❑	❑
Ich hatte keine Lust an der Sporttherapie teilzunehmen	❑	❑	❑	❑
Das Programm stellte eine positive Abwechslung zum Klinikalltag dar	❑	❑	❑	❑
Die Sporttherapie hat mir Spaß gemacht	❑	❑	❑	❑
Das Sportangebot war an meine Bedürfnisse angepasst	❑	❑	❑	❑
Die Übungen waren abwechslungsreich	❑	❑	❑	❑

Während der Intensivtherapie erhalten einige Kinder/Jugendliche ergänzend zur Sporttherapie eine physiotherapeutische Behandlung. Uns interessiert nun, ob Du sowohl Sport- als auch Physiotherapie erhalten hast und wie Du die Kombination empfunden hast.

6. Hast Du während Deines stationären Aufenthaltes ergänzend zur Sporttherapie eine physiotherapeutische Behandlung erhalten?
Kreuze die zutreffende Antwort an!

❑ Weiß ich nicht

❑ Nein

❑ Ja

7. Wenn Du die vorherige Frage mit Ja beantwortet hast, interessiert uns, wie Du die Kombination aus der Sport- und Physiotherapie wahrgenommen hast.

Die Kombination aus Sport- und Physiotherapie...
Kreuze die zutreffenden Antworten an!

❑ ... hat mir gutgetan

❑ ... war eine gute Ergänzung

❑ ... war gut aufeinander abgestimmt

❑ ... war für mich genau gleich

❑ ... war mir zu viel

❑ Sonstiges: ____________________

8. Abschließend möchten wir von Dir noch gerne wissen, ob Du uns zum sporttherapeutischen Angebot etwas rückmelden möchtest?
Fülle gegebenenfalls bitte die Kästchen aus!

Das hätte ich mir noch gewünscht (Verbesserungsvorschläge):

Das hat mir besonders gut gefallen:

Sonstiges:

Datum: _ _ / _ _ / _ _ _ _

Vielen lieben Dank für Deine Teilnahme und Angaben, Du hast uns damit wirklich sehr geholfen!

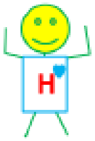

2. Satisfaction survey – 2025

2.1. 2-6 years

HELD*INNEN IN BEWEGUNG

UNIVERSITÄTS KLINIKUM FREIBURG
KJK KINDER- UND JUGENDKLINIK

Zufriedenheitsfragebogen – Sporttherapie 2-6 Jahre

Liebe Eltern,

seit März 2020 gibt es eine Sport- und Bewegungstherapie auf den Stationen der Pädiatrischen Hämatologie und Onkologie in der Kinder- und Jugendklinik (KJK) des Universitätsklinikums Freiburg. Mit dem Ziel, unser Sportprogramm stetig zu verbessern und an die Bedürfnisse Ihres Kindes anzupassen, möchten wir über einen Fragebogen herausfinden, wie zufrieden Sie mit dem aktuellen Sportangebot für Ihr Kind sind. Die Beantwortung der Fragen ist freiwillig. Wenn Sie möchten, können Sie den Fragebogen gerne zusammen mit Ihrem Kind ausfüllen. Am Ende stellen wir auch noch einige Fragen an Sie als Eltern. Insgesamt wird das Ausfüllen ca. 10 Minuten dauern.

Hinweise zum Ausfüllen des Fragebogens:

- Wir sind an Ihrer Meinung in Bezug auf Ihre Zufriedenheit mit der Sporttherapie für Ihr Kind interessiert, deshalb gibt es keine „richtigen“ oder „falschen“ Antworten.
- Falls eine Frage weniger auf Ihr Kind zutrifft oder es Ihnen schwerfällt, sich für eine Antwort zu entscheiden, kreuzen Sie bitte die Antwort an, die Ihnen spontan am passendsten erscheint.
- Bitte kreuzen Sie die für Ihr Kind zutreffenden Kästchen an: ☒
- Fragen, bei denen wir Sie bitten, etwas aufzuschreiben, sind durch einen Kasten: [ ] gekennzeichnet.
- Der Fragebogen wird anonymisiert ausgewertet, wir können Ihre Angaben im Fragebogen also nicht mit Ihrem Namen in Verbindung bringen.

Vielen Dank und viel Spaß beim Ausfüllen!

Mit freundlichen Grüßen

Ihr Sportteam ☺

1. Wer füllt den Fragebogen aus?
Bitte Zutreffendes ankreuzen.

☐ Elternteil/Bezugsperson mit Kind
☐ Elternteil/Bezugsperson alleine

Als Erstes möchten wir gerne von Ihnen wissen, wie Ihrem Kind die Sporttherapie hier in der Klinik gefällt und wie wir Sporttherapeut*innen das Angebot noch besser gestalten können.

2. Wie zufrieden ist Ihr Kind mit …
Bitte Zutreffendes ankreuzen.

	Sehr zufrieden	Eher zufrieden	Eher unzufrieden	Ziemlich unzufrieden
… dem Sportangebot insgesamt?	☐	☐	☐	☐
… dem Ablauf (z.B. der Kontaktaufnahme)?	☐	☐	☐	☐
… den Inhalten (z.B. Übungsauswahl)?	☐	☐	☐	☐
… der Häufigkeit?	☐	☐	☐	☐
… der Dauer?	☐	☐	☐	☐
… den Räumen, in denen die Sporttherapie stattfindet?	☐	☐	☐	☐
… mit den Sportmaterialien? 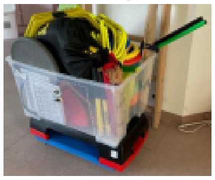	☐	☐	☐	☐

HELD*INNEN IN BEWEGUNG

UNIVERSITÄTS KLINIKUM FREIBURG
KJK KINDER- UND JUGENDKLINIK

3. Inwieweit treffen folgende Aussagen zur Sporttherapie auf Ihr Kind zu?
Bitte Zutreffendes ankreuzen.

	Trifft voll zu	Trifft zu	Trifft nicht zu	Trifft überhaupt nicht zu
Das Sportprogramm ist an den aktuellen Gesundheitszustand meines Kindes angepasst.	☐	☐	☐	☐
Die Sporttherapie während des stationären Aufenthaltes tut meinem Kind gut.	☐	☐	☐	☐
Die Sporttherapie macht meinem Kind Spaß.	☐	☐	☐	☐
Mein Kind hat keine Lust an der Sporttherapie teilzunehmen.	☐	☐	☐	☐
Das Sportangebot ist an die Bedürfnisse meines Kindes angepasst.	☐	☐	☐	☐
Die Sportspiele/Übungen sind abwechslungsreich.	☐	☐	☐	☐
Die Sporttherapie soll häufiger stattfinden.	☐	☐	☐	☐
Bei der Sporttherapie fühlt sich mein Kind sicher.	☐	☐	☐	☐
Mein Kind würde einem Freund/einer Freundin das Sportprogramm empfehlen, wenn er/sie in einer ähnlichen Situation wie mein Kind wäre.	☐	☐	☐	☐

4. Wo führt Ihr Kind während stationärer Aufenthalte gerne die Sporttherapie durch?
Bitte Zutreffendes ankreuzen. Mehrere Angaben sind möglich.

☐ Im Stationszimmer
☐ Auf dem Stationsflur
☐ In einem Sportraum
☐ Ist überall möglich

5. Wann ist für Ihr Kind die beste Zeit während stationärer Aufenthalte, um an der Sporttherapie teilzunehmen?
Bitte Zutreffendes ankreuzen.

☐ Morgens
☐ Mittags
☐ Abends
☐ Ist meinem Kind egal

6. Wie viele Stunden Sport pro Tag würde Ihr Kind gerne machen?
Setzen Sie auf dem Zeitstrahl ein passendes Kreuz bei Ihrer gewünschten Zahl. Treibt Ihr Kind z.B. 1,5 Stunden pro Tag gerne Sport, platzieren Sie das Kreuz zwischen der 1 & 2.

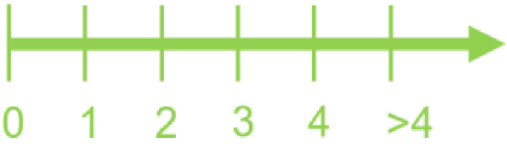

7. Würde Ihr Kind gerne in der Zeit zuhause, wenn sie nicht im Krankenhaus sind, Sport machen?
Bitte Zutreffendes ankreuzen. Mehrere Antworten sind möglich.

☐ Ja, am liebsten zuhause mit einer/m Sporttherapeut/in
☐ Ja, am liebsten in einer kleinen Gruppe
☐ Ja, am liebsten Online mit den Sporttherapeut*innen aus der Klinik
☐ Nein, kein Interesse
☐ Ist uns egal

Sonstiges:

HELD*INNEN IN BEWEGUNG

UNIVERSITÄTS KLINIKUM FREIBURG
KJK KINDER- UND JUGENDKLINIK

8. **Abschließend möchten wir von Ihnen noch gerne wissen, ob Sie uns für Ihr Kind zum sporttherapeutischen Angebot etwas rückmelden möchten?**
   Bitte füllen Sie die grauen Felder aus.

Das gefällt mir für mein Kind an der Sporttherapie besonders gut:

Das gefällt mir an der Sporttherapie für mein Kind nicht so gut:

Das würde ich mir für mein Kind noch wünschen:

Sonstige Anmerkungen:

Vielen Dank für Ihre Teilnahme! Damit haben Sie uns sehr geholfen! ☺

Datum: _ _ / _ _ / _ _ _ _

2.2 7-11 years

HELD*INNEN IN BEWEGUNG

UNIVERSITÄTS KLINIKUM FREIBURG
KJK KINDER- UND JUGENDKLINIK

Zufriedenheitsfragebogen – Sporttherapie 7-11 Jahre

Liebe Kinder und liebe Eltern,

seit März 2020 gibt es eine Sport- und Bewegungstherapie auf den Stationen der Pädiatrischen Hämatologie und Onkologie in der Kinder- und Jugendklinik (KJK) des Universitätsklinikums Freiburg. Mit dem Ziel, unser Sportprogramm stetig zu verbessern und an Deine Bedürfnisse anzupassen, möchten wir über einen Fragebogen herausfinden, wie zufrieden Du mit dem aktuellen Sportangebot bist. Die Beantwortung der Fragen ist freiwillig. Wenn Du möchtest, kannst Du den Fragebogen gerne zusammen mit Deinen Eltern ausfüllen. Am Ende stellen wir auch noch einige Fragen an Sie als Eltern. Insgesamt wird das Ausfüllen ca. 10 Minuten dauern.

Hinweise zum Ausfüllen des Fragebogens:

- Wir sind an Deiner Meinung in Bezug auf Deine Zufriedenheit interessiert, deshalb gibt es keine „richtigen" oder „falschen" Antworten.
- Falls eine Frage weniger auf Dich zutrifft oder es Dir schwerfällt, Dich für eine Antwort zu entscheiden, kreuze bitte die Antwort an, die Dir spontan am passendsten erscheint.
- Bitte kreuze die für Dich zutreffenden Kästchen bzw. Smileys an: ☒
- Fragen, bei denen wir Dich bitten, etwas aufzuschreiben, sind durch einen Kasten: ▭ gekennzeichnet.
- Der Fragebogen wird anonymisiert ausgewertet, wir können Deine Angaben im Fragebogen also nicht mit Deinem Namen in Verbindung bringen.

Vielen Dank und viel Spaß beim Ausfüllen!

Mit freundlichen Grüßen

Dein Sportteam ☺

HELD*INNEN IN BEWEGUNG

UNIVERSITÄTS KLINIKUM FREIBURG
KJK KINDER- UND JUGENDKLINIK

1. **Wer füllt den Fragebogen aus?**
   Bitte Zutreffendes ankreuzen.

☐ Kind alleine

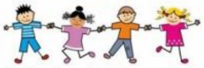

☐ Kind mit Elternteil/Bezugsperson

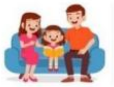

Als Erstes möchten wir gerne von Dir wissen, wie Dir die Sporttherapie hier in der Klinik gefällt und wie wir Sporttherapeut*innen das Angebot noch besser gestalten können.

2. **Wie zufrieden bist Du mit …**
   Kreuze den passenden Smiley an.

		Sehr zufrieden	Eher zufrieden	Eher unzufrieden	Ziemlich unzufrieden
… dem Sportangebot insgesamt?	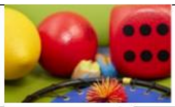				
… dem Ablauf (z.B. der Kontakt-aufnahme)?	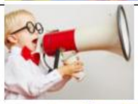				
… den Inhalten (z.B. Übungs-auswahl)?	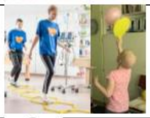				
… der Häufigkeit?	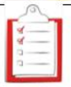				
… der Dauer?	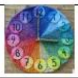				
… den Räumen, in denen die Sporttherapie stattfindet?	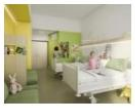				
… mit den Sportmaterialien?	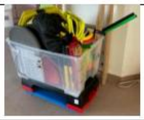				

3. Inwieweit treffen folgende Aussagen zur Sporttherapie auf Dich zu?
Kreuze den passenden Smiley an.

		Trifft voll zu	Trifft zu	Trifft nicht zu	Trifft gar nicht zu
Das Sportangebot passt gut zu mir.	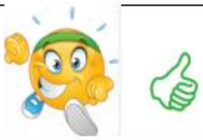				
Sport im Krankenhaus tut mir gut.	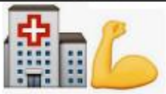				
Die Sporttherapie sorgt für Abwechslung in meinem Alltag.	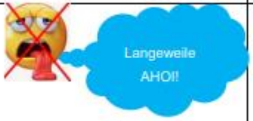				
Sporttherapie macht mir Spaß.	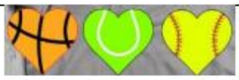				
Ich habe keine Lust auf Sporttherapie.	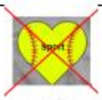				
Beim Sport machen wir das worauf ich Lust habe.	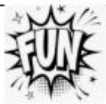				
Ich fühle mich gut beim Sport.	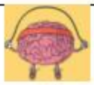				
Die Sportspiele sind abwechslungsreich.	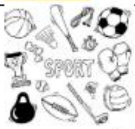				
Ich würde gerne öfters an der Sporttherapie mitmachen.	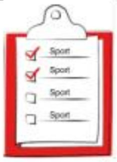				
Bei der Sporttherapie fühle ich mich sicher.	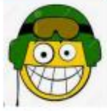				
Andere Kinder würde ich motivieren, an der Sporttherapie mitzumachen.	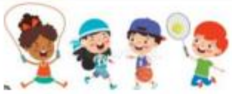				

HELD*INNEN IN BEWEGUNG

UNIVERSITÄTS KLINIKUM FREIBURG
KJK KINDER- UND JUGENDKLINIK

4. **Wo würdest Du die Sporttherapie im Krankenhaus am liebsten machen?**
Bitte Zutreffendes ankreuzen. Mehrere Angaben sind möglich.

☐ Im Stationszimmer

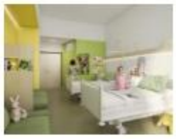

☐ Auf dem Stationsflur

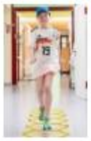

☐ In einem Sportraum

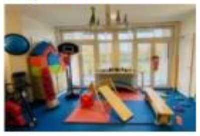

☐ Passt überall

5. **Wann machst Du am liebsten Sport, wenn Du im Krankenhaus bist?**
Bitte Zutreffendes ankreuzen.

☐ Morgens

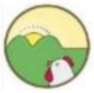

☐ Mittags

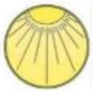

☐ Abends

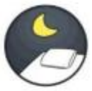

☐ Ist mir egal

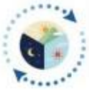

6. **Wie viele Stunden pro Tag würdest Du gerne Sport machen?**
Setze auf dem Zeitstrahl ein passendes Kreuz bei Deiner gewünschten Zahl. Treibst Du z.B. gerne 1,5 Stunden pro Tag Sport, platziere das Kreuz zwischen der 1 & 2; > 4 = mehr als 4 Stunden pro Tag.

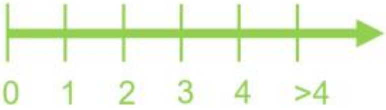

7. **Würdest Du gerne in der Zeit zuhause, wenn Du nicht im Krankenhaus bist, Sport machen?**
Bitte Zutreffendes ankreuzen. Mehrere Antworten sind möglich.

☐	Ja, am liebsten zuhause mit einer/m Sporttherapeut/in	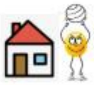
☐	Ja, am liebsten in einer kleinen Gruppe	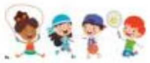
☐	Ja, am liebsten Online mit den Sporttherapeut*innen aus der Klinik	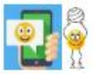
☐	Nein, ich habe kein Interesse	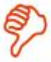
☐	Ist mir egal	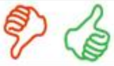

Sonstiges:

8. **Abschließend möchten wir von Dir noch gerne wissen, ob Du uns zum sporttherapeutischen Angebot etwas rückmelden möchtest?**
   Bitte schreibe etwas in die grauen Felder.

Das gefällt mir an der Sporttherapie besonders gut:

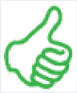

Das mag ich an der Sporttherapie nicht so gerne:

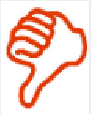

Das würde ich mir noch wünschen:

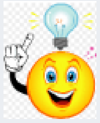

Sonstige Anmerkungen:

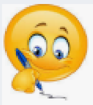

Vielen Dank für Deine Teilnahme! Damit hast Du uns wirklich sehr geholfen!

Datum: _ _ / _ _ / _ _ _ _

2.3 12 years and older

HELD*INNEN IN BEWEGUNG

UNIVERSITÄTS KLINIKUM FREIBURG
KJK KINDER- UND JUGENDKLINIK

Zufriedenheitsfragebogen – Sporttherapie ab 12 Jahre

Liebe Kinder, Jugendliche und liebe Eltern,

seit März 2020 gibt es eine Sport- und Bewegungstherapie auf den Stationen der Pädiatrischen Hämatologie und Onkologie in der Kinder- und Jugendklinik (KJK) des Universitätsklinikums Freiburg. Mit dem Ziel, unser Sportprogramm stetig zu verbessern und an Deine Bedürfnisse anzupassen, möchten wir über einen Fragebogen herausfinden, wie zufrieden Du mit dem aktuellen Sportangebot bist. Die Beantwortung der Fragen ist freiwillig. Wenn Du möchtest, kannst Du den Fragebogen gerne zusammen mit Deinen Eltern ausfüllen. Am Ende stellen wir auch noch einige Fragen an Sie als Eltern. Insgesamt wird das Ausfüllen ca. 10 Minuten dauern.

Hinweise zum Ausfüllen des Fragebogens:

- Wir sind an Deiner Meinung in Bezug auf Deine Zufriedenheit interessiert, deshalb gibt es keine „richtigen" oder „falschen" Antworten.
- Falls eine Frage weniger auf Dich zutrifft oder es Dir schwerfällt, Dich für eine Antwort zu entscheiden, kreuze bitte die Antwort an, die Dir spontan am passendsten erscheint.
- Bitte kreuze die für Dich zutreffenden Kästchen an: ☒
- Fragen, bei denen wir Dich bitten, etwas aufzuschreiben, sind durch einen Kasten: [ ] gekennzeichnet.
- Der Fragebogen wird anonymisiert ausgewertet, wir können Deine Angaben im Fragebogen also nicht mit Deinem Namen in Verbindung bringen.

Vielen Dank und viel Spaß beim Ausfüllen!

Mit freundlichen Grüßen

Dein Sportteam ☺

1. **Wer füllt den Fragebogen aus?**
   Bitte Zutreffendes ankreuzen.

☐ Kind/Jugendliche(r) alleine
☐ Kind/Jugendliche(r) mit Elternteil/Bezugsperson

Als Erstes möchten wir gerne von Dir wissen, wie Dir die Sporttherapie hier in der Klinik gefällt und wie wir Sporttherapeut*innen das Angebot noch besser gestalten können.

2. **Wie zufrieden bist Du mit …**
   Bitte Zutreffendes ankreuzen.

	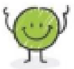 Sehr zufrieden	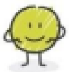 Eher zufrieden	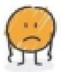 Eher unzufrieden	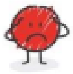 Ziemlich unzufrieden
… dem Sportangebot insgesamt?	☐	☐	☐	☐
… dem Ablauf (z.B. der Kontaktaufnahme)?	☐	☐	☐	☐
… den Inhalten (z.B. Übungsauswahl)?	☐	☐	☐	☐
… der Häufigkeit?	☐	☐	☐	☐
… der Dauer?	☐	☐	☐	☐
… den Räumen, in denen die Sporttherapie stattfindet?	☐	☐	☐	☐
… mit den Sportmaterialien? 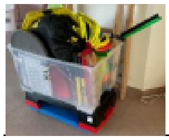	☐	☐	☐	☐

HELD*INNEN IN BEWEGUNG

UNIVERSITÄTS KLINIKUM FREIBURG
KJK KINDER- UND JUGENDKLINIK

3. Inwieweit treffen folgende Aussagen zur Sporttherapie auf Dich zu?
Bitte Zutreffendes ankreuzen.

	Trifft voll zu	Trifft zu	Trifft nicht zu	Trifft überhaupt nicht zu
Das Sportprogramm ist an meinen aktuellen Gesundheitszustand angepasst.	☐	☐	☐	☐
Ich habe das Gefühl, dass mir der Sport während des stationären Aufenthaltes guttut.	☐	☐	☐	☐
Das Bewegungsprogramm ist eine positive Abwechslung zum Klinikalltag für mich.	☐	☐	☐	☐
Die Sporttherapie macht mir Spaß.	☐	☐	☐	☐
Ich habe keine Lust an der Sporttherapie teilzunehmen.	☐	☐	☐	☐
Ich erhalte die Form der Bewegung, die ich möchte.	☐	☐	☐	☐
Das Sportangebot ist an meine Bedürfnisse angepasst.	☐	☐	☐	☐
Die Spiele/Übungen sind abwechslungsreich.	☐	☐	☐	☐
Die Sporttherapie soll häufiger stattfinden.	☐	☐	☐	☐
Bei der Sporttherapie fühle ich mich sicher.	☐	☐	☐	☐
Ich würde einem Freund/einer Freundin das Sportprogramm empfehlen, wenn er/sie in einer ähnlichen Situation wie ich wäre.	☐	☐	☐	☐

4. **Wo würdest Du während stationärer Aufenthalte gerne die Sporttherapie durchführen?**
   Bitte Zutreffendes ankreuzen. Mehrere Angaben sind möglich.

☐ In meinem Zimmer
☐ Auf dem Stationsflur
☐ In einem Sportraum
☐ Ist überall möglich

5. **Wann ist für Dich die beste Zeit während stationärer Aufenthalte, um an der Sporttherapie teilzunehmen?**
   Bitte Zutreffendes ankreuzen.

☐ Morgens
☐ Mittags
☐ Abends
☐ Ist mir egal

6. **Wie viele Stunden pro Tag würdest Du gerne Sport machen?**
   Setze auf dem Zeitstrahl ein Kreuz bei Deiner gewünschten Zahl. Machst Du z.B. 1,5 Stunden pro Tag gerne Sport, platziere Dein Kreuz zwischen der 1 & 2.

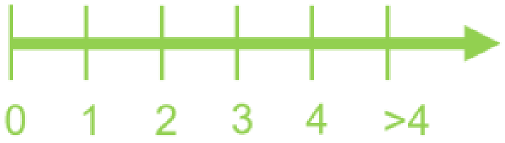

7. **Würdest Du gerne in der Zeit zuhause, wenn Du nicht im Krankenhaus bist, Sport machen?**
   Bitte Zutreffendes ankreuzen. Mehrere Antworten sind möglich.

☐ Ja, am liebsten zuhause mit einer/m Sporttherapeut/in
☐ Ja, am liebsten in einer kleinen Gruppe
☐ Ja, am liebsten Online mit den Sporttherapeut*innen aus der Klinik
☐ Nein, ich habe kein Interesse
☐ Ist mir egal

Sonstiges:

8. **Abschließend möchten wir von Dir noch gerne wissen, ob Du uns zum sporttherapeutischen Angebot etwas rückmelden möchtest?**
Bitte fülle die grauen Felder aus.

Das gefällt mir an der Sporttherapie besonders gut:

Das mag ich an der Sporttherapie nicht so gerne:

Das würde ich mir noch wünschen:

Sonstige Anmerkungen:

Vielen Dank für Deine Teilnahme! Damit hast Du uns wirklich sehr geholfen! ☺

Datum: _ _ / _ _ / _ _ _ _

2.4. Parental survey

HELD*INNEN IN BEWEGUNG

UNIVERSITÄTS KLINIKUM FREIBURG
KJK KINDER- UND JUGENDKLINIK

Zufriedenheitsfragebogen für die Eltern

Liebe Eltern,

im Rahmen einer Zufriedenheitsanalyse möchten wir herausfinden, wie zufrieden Sie als Elternteil/Bezugsperson mit dem sporttherapeutischen Angebot in der Pädiatrischen Hämatologie und Onkologie der Kinder- und Jugendklinik (KJK) in Freiburg sind. In Zukunft möchten wir das Bewegungsprogramm für die gesamte Familie integrativer gestalten. Hierzu würden wir uns über Ihre Meinung und Erfahrungen mit dem Sportangebot freuen.

Hinweise zum Ausfüllen des Fragebogens:

- Wir sind an Ihrer Meinung in Bezug auf Ihre Zufriedenheit mit der Sporttherapie interessiert, deshalb gibt es keine „richtigen“ oder „falschen“ Antworten.
- Falls eine Frage weniger auf Sie zutrifft oder es Ihnen schwerfällt, sich für eine Antwort zu entscheiden, kreuzen Sie bitte die Antwort an, die Ihnen spontan am passendsten erscheint.
- Bitte kreuzen Sie die zutreffenden Kästchen an: ☒
- Fragen, bei denen wir Sie bitten, etwas aufzuschreiben, sind durch einen Kasten: [ ] gekennzeichnet.
- Der Fragebogen wird anonymisiert ausgewertet, wir können Ihre Angaben im Fragebogen also nicht mit Ihrem Namen in Verbindung bringen.

Vielen Dank und viel Spaß beim Ausfüllen!

Mit freundlichen Grüßen

Ihr Sportteam ☺

HELD*INNEN IN BEWEGUNG

UNIVERSITÄTS KLINIKUM FREIBURG
KJK KINDER- UND JUGENDKLINIK

1. **Würden Sie gerne während der stationären Aufenthalte an einem Sportangebot für Eltern teilnehmen?**
   Bitte Zutreffendes ankreuzen.

☐ Ja → *weiter zu Frage 2*
☐ Nein → *weiter zu Frage 3*

2. **Unter welchen Rahmenbedingungen können Sie sich die Teilnahme an der Sporttherapie während stationärer Aufenthalte vorstellen?**
   Bitte Zutreffendes ankreuzen.

	Trifft voll zu	Trifft zu	Trifft nicht zu	Trifft überhaupt nicht zu
… gemeinsam mit meinem Kind.	☐	☐	☐	☐
… in 1:1 Betreuung.	☐	☐	☐	☐
… gemeinsam mit meinem Kind und anderen Familien.	☐	☐	☐	☐
… während mein Kind anderweitig beschäftigt ist mit anderen Eltern.	☐	☐	☐	☐
**Ort**				
… auf dem Stationsflur.	☐	☐	☐	☐
… in einem Sportraum.	☐	☐	☐	☐
… bei schönem Wetter draußen (z.B. auf der Terrasse).	☐	☐	☐	☐
… im Elternhaus.	☐	☐	☐	☐

Sonstiges:

3. **Wünschen Sie sich, dass Geschwisterkinder mehr in die Sporttherapie eingebunden werden?**
   Bitte Zutreffendes ankreuzen.

☐ Ja
☐ Nein
☐ Keine Angabe, da keine Geschwister in der Familie

Sonstiges:

4. Rückmeldung zur stationären Sporttherapie:

Das gefällt mir an der Sporttherapie besonders gut:

Das mag ich an der Sporttherapie nicht so gerne:

Das würde ich mir noch wünschen:

Sonstige Anmerkungen:

HELD*INNEN IN BEWEGUNG

UNIVERSITÄTS KLINIKUM FREIBURG
KJK KINDER- UND JUGENDKLINIK

Zukünftig ist ein zentrumsübergreifendes Projekt im Rahmen des Netzwerks ActiveOncoKids (NAOK) zur Erfassung unerwünschter Ereignisse (AE: Adverse Events) während der Sporttherapie geplant, um Sporteinheiten in Kliniken noch sicherer zu gestalten.

5. **Kennen Sie bereits das Netzwerk ActiveOncoKids (NAOK) inklusive Angebot und Newsletter?**
   Bitte Zutreffendes ankreuzen und ggfs. ergänzen.

☐ Ja
☐ Nein
☐ Nein, aber ich nutze folgende (Online-)Quellen:

6. **Könnten Sie sich vorstellen, an diesem Projekt für das Eintreten von unerwünschten Vorfällen, die während den Sporteinheiten mit Ihrem Kind auftreten könnten, teilzunehmen?**
   Bitte Zutreffendes ankreuzen und ggfs. begründen.

☐ Ja
☐ Nein → aus welchem Grund?

7. **Finden Sie solche Projekte grundsätzlich sinnvoll und wichtig?**
   Bitte Zutreffendes ankreuzen und ggfs. begründen.

☐ Ja
☐ Nein → aus welchem Grund?

Vielen Dank für Ihre Teilnahme ☺

Datum: _ _ / _ _ / _ _ _ _
