# Supplementary material for: Heroes in motion – a six-year quality report and patient evaluation of a real-world exercise therapy program in pediatric oncology
Source: Front Pediatr. 2026 Jun 9;14:1819559. doi: 10.3389/fped.2026.1819559 (PMC13286956; doi:10.3389/fped.2026.1819559)
Supplement: Supplementary file 2 [file Datasheet2.pdf]

TIDieR Checklist – HIM-Program – Exercise therapy in pediatric hematology & oncology

TIDieR Item	Description
**1. Brief name**	HIM (Heroes in Motion) program within pediatric exercise oncology care
**2. Why (rationale)**	To counteract physical inactivity, maintain functional capacity, reduce treatment-related side effects, and promote psychosocial well-being in children and adolescents with hematological and oncological diseases, particularly during and after intensive treatments such as HSCT
**3. What (materials)**	Exercise equipment adapted to pediatric oncology patients; ward-based materials; individualized home exercise plans (supplementary materials)
**4. What (procedures)**	Individualized and supervised exercise therapy including strength, endurance, coordination, flexibility, sensorimotor, and play-based activities; outpatient sessions follow standardized structure (warm-up, main phase, cool-down); inpatient delivery is condition-dependent and flexible
**5. Who provided**	One to two certified exercise therapists with expertise in pediatric oncology under continuous clinical supervision
**6. How**	Face-to-face delivery in inpatient wards and outpatient facility; individual and small-group formats; family involvement; integration of community-based exercise events
**7. Where**	Inpatient: bedside, patient rooms, ward corridors in a tertiary pediatric oncology center; Outpatient: exercise room of the parents' house of the "Förderverein für krebskranke Kinder e.V."
**8. When and how much**	Inpatient: flexible weekday delivery (approximately 3–5 sessions/week depending on staffing and clinical feasibility); Outpatient: fixed twice-weekly sessions (30–60 min); overall frequency dependent on clinical course (2020–2025 evaluation period)
**9. Tailoring (personalization)**	Continuous adaptation to medical status, treatment phase, developmental level, and physical/psychological resilience; modification or pause in case of contraindications (e.g., thrombocytopenia, anemia, fever, infections, acute symptoms)
**10. Modifications over time**	Expansion from inpatient-only (2020) to inclusion of outpatient program (2023); increased training frequency and community-based formats; temporary staffing reduction (Oct 2024–Sep 2025) with reduced continuity of care; full resumption from Oct 2025
**11. Planned vs actual delivery**	Real-world implementation embedded in routine care; no fixed session prescription per patient; delivery dependent on staffing, clinical feasibility, and patient condition; weekend sessions not routinely provided
**12. Fidelity (planned vs delivered consistency)**	Inpatient delivery flexible and opportunistic; outpatient delivery standardized; adherence ensured through qualified staff and clinical supervision; variability reflects routine-care implementation rather than protocolized trial conditions
