# Supplementary material for: Heroes in motion – a six-year quality report and patient evaluation of a real-world exercise therapy program in pediatric oncology
Source: Front Pediatr. 2026 Jun 9;14:1819559. doi: 10.3389/fped.2026.1819559 (PMC13286956; doi:10.3389/fped.2026.1819559)
Supplement: Supplementary file 3 [file Datasheet3.pdf]

Supplementary Material

Home-based exercise plan:

BEWEGUNGSPLAN

**MOBILISATION**	**Hinweise/Ausführung**	**Dauer/Anzahl/Wiederholung**
**„Wischen"-seitlicher Ausfallschritt** (auf einem Handtuch etc.): 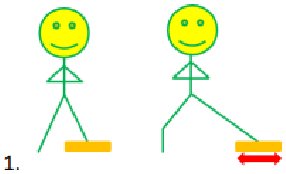 1.	Bewegungsausführung erfolgt stets aus den Beinen, Oberkörper bleibt aufrecht & stabil (evtl. an einer Wand zur Unterstützung stabilisieren):<br>1. Das Standbein wird gebeugt und das „wischende" Bein wird geradlinig in die Streckung geführt<br><br>Hinweis: Nur so weit „wischen", damit Du aus eigener Beinkraft wieder in die Ausgangsposition zurückgelangen kannst, ohne dabei Ausgleichbewegungen mit dem Oberkörper durchzuführen	3 x 5-20 Wdhl. pro Seite, 10s Pause zum Beinwechsel
**„Wischen"-Ausfallschritt:** 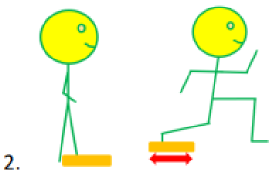 2.	2. Ausfallschritt: Das vordere Bein geht in die Beugung, während das Bein auf dem Handtuch geradlinig nach hinten „wischt" (gegengleicher Armeinsatz)	3 x 5-20 Wdhl. pro Seite, 10s Pause zum Beinwechsel
**Hampelmann:** 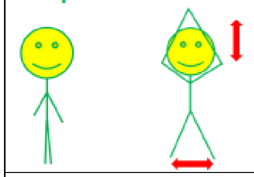	• Während die Beine auseinanderspringen und die Knie leicht gebeugt werden, klatschen die Hände überkopf zusammen<br>• Variation: Die Arme bewegen sich wie zuvor, wohingegen mit den Beinen Wechselsprünge durchgeführt werden	3 x 5-20 Wdhl.
**GLEICHGEWICHTSSCHULUNG**		
**Einbeinstand:** 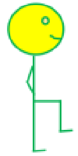	• Schuhe ausziehen, evtl. an einer Wand zur Unterstützung stabilisieren<br>• Vorstufe Einbeinstand: 1. 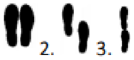 2. 3.<br>• Hände an die Hüfte<br>Einbeinstand:<br>• Ein Fuß vom Boden lösen, hüfthohes Knie (Spielbein), Standbein leicht gebeugt<br>• Variation: instabiler Untergrund (Handtuch), Augen schließen, Ball werfen	3 x 20s links/rechts, 10s Pause zum Beinwechsel

Carolin Ohnmacht (M.Sc. Sportwissenschaft u. Sporttherapie, carolin.ohnmacht@uniklinik-freiburg.de)

**KRÄFTIGUNGSÜBUNGEN**		
**Balltransport auf dem Rücken:** 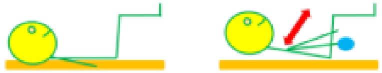	Kräftigung der Bauchmuskulatur:<br>• Liegende Sitzposition einnehmen (Unterschenkel parallel zum Boden)<br>• Ball/Gegenstand unter den Beinen von der einen in die andere Hand übergeben	3 x 5-20 x links-/rechtsherum
**Brücke:** 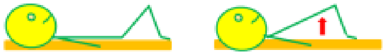	• Ausgangsposition: Rücklage einnehmen, Knie sind angewinkelt & die Füße aufgestellt, die Arme liegen entspannt neben dem Körper<br>• Hüfte vom Boden nach oben abheben, nur die Füße & Schulterblätter haben Untergrundkontakt (Oberkörper und die Oberschenkel bilden eine gerade Linie)	3 x 10-30s<br>Oder: 3 x 5-20 Wdhl. (Hüfte abwechselnd heben & senken → nicht komplett)
**Wand-Sitz:** 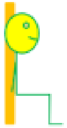	Kräftigung der Oberschenkelmuskulatur:<br>• 90°-Hockposition einnehmen<br>• Oberkörper in Kontakt zur Wand<br>• Beine hüftbreit aufstellen; Füße zeigen gerade nach vorne<br>Variation: Zusätzlich (mit einem Partner) einen Ball hin- und herwerfen	3 x 10-30s
**Wand-Liegestütz:** 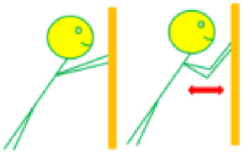	Kräftigung der Arm- und Brustmuskulatur:<br>• Hände etwas breiter als schulterbreit & auf Brusthöhe platzieren<br>• Beine sind gestreckt, Körper bildet eine gerade Linie<br>• Bewegungsausführung: Körper in Richtung Hände führen, Arme beugen, Nasenspitze Richtung Wand	3 x 5-20 Wdhl.
**Zehenspitzenstand:** 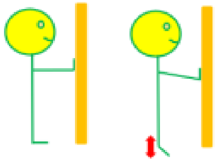	Kräftigung der Wadenmuskulatur:<br>• Zehenspitzenstand einnehmen (evtl. an einer Wand zur Unterstützung stabilisieren) → Position ca. 2-3s halten, danach wieder absenken<br>• Kurz bevor die Ferse auf dem Boden aufsetzt, Bewegung erneut einleiten und maximal in die Streckung gehen	3 x 5-20 Wdhl.
**BEWEGLICHKEIT**		
**Wirbel-Rollen:** 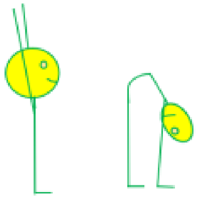	Dehnung der Oberschenkelrückseite & des Rückenstreckers:<br>• Ausgangsposition: Stabiler, aufrechter Stand, Arme sind nach oben gestreckt (evtl. Zehenspitzposition einnehmen)<br>• Durchführung: Wirbel für Wirbel den Oberkörper abrollen und mit den Fingerspitzen in Richtung Füße bewegen → Position 2-3s halten, dann wieder langsam und gleichmäßig nach oben aufrollen	3-5 x

Carolin Ohnmacht (M.Sc. Sportwissenschaft u. Sporttherapie, carolin.ohnmacht@uniklinik-freiburg.de)

R U M P F S T A B I L I S A T I O N S – T R A I N I N G:

**Übungsdauer:**	Beginnend mit 20s, wöchentlich um 5s steigern bis 45s erreicht sind
**Wechselzeit/Pause zwischen den Übungen:**	5-15s
**Wiederholung des Zirkels:**	3x
**Häufigkeit/Woche:**	3-4x wäre optimal

Übungsreihenfolge:

Übung	Hinweis	Variation:
1. **Unterarmstütz** 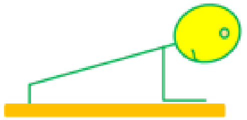	Körper bildet eine gerade Linie (kein Hohlkreuz), Kopf in Verlängerung der Wirbelsäule (WS), Bauch anspannen (Bauchnabel in Richtung WS ziehen), Ellbogen- unterhalb des Schultergelenks platzieren	- Bein im Wechsel vom Boden abheben<br>- Vor-/zurückwippen (aus dem Schultergelenk)<br>- Seitlich mit der Hüfte kippen
2. **Bauch** (Crunches) 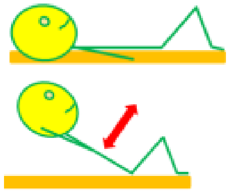	Ausgangsposition: Rücklage einnehmen, Beine im 90°-Winkel aufstellen oder Hockerposition (siehe oben) einnehmen<br><br>Durchführung: Hände z.B. seitlich an den Ohren platzieren, Oberkörper bildet mit dem Kopf eine Linie, kurz bevor die Schulterblätter aufliegen Bewegung erneut einleiten<br><br>Bei der Bewegungsausführung aus- und bei der Entspannung einatmen	- Hockerposition: Ball durchkreisen (siehe oben)<br>- Radfahrbewegung<br>- Käfer (diagonal: 1 Arm + 1 Bein im Wechsel ausstrecken)
3. + 4. **Seitstütz** (li/re) 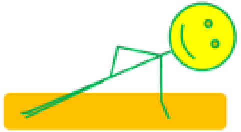	Füße aufeinanderlegen, Ellbogen unter dem Schultergelenk im 90°-Winkel platzieren<br><br>Vorstufe: Knie liegen auf dem Boden (90° Kniewinkel)<br>Fortgeschritten: Nur die Füße und der Stützarm haben Bodenkontakt (siehe Bild)	- Bein kurz abheben<br>- Mit der Hüfte wippen
5. **Rücken** (Bauchlage) 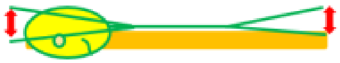	Bauchlage einnehmen, Kopf in Verlängerung der WS, Beine und Arme vom Boden abheben	- Mit den Armen & Beinen hacken/paddeln<br>- Ball mit den Armen herumkreisen

Carolin Ohnmacht (M.Sc. Sportwissenschaft u. Sporttherapie, carolin.ohnmacht@uniklinik-freiburg.de)
